# Radiation dose and fluoroscopy time of aneurysm coiling in patients with unruptured and ruptured intracranial aneurysms as a function of aneurysm size, location, and patient age

**DOI:** 10.1007/s00234-022-03092-8

**Published:** 2022-11-22

**Authors:** Marcel Opitz, Celina Zenk, Sebastian Zensen, Denise Bos, Yan Li, Hanna Styczen, Marvin Darkwah Oppong, Ramazan Jabbarli, Tim Hagenacker, Michael Forsting, Isabel Wanke, Cornelius Deuschl

**Affiliations:** 1 Institute of Diagnostic and Interventional Radiology and Neuroradiology, Faculty of Medicine, University Hospital Essen, Hufelandstrasse 55, 45147 Essen, Germany; 2 Department of Neurosurgery and Spine Surgery and Center for Translational Neuro- and Behavioral Science (C-TNBS), University Hospital Essen, Essen, Germany; 3 Department of Neurology and Center for Translational Neuro- and Behavioral Science (C-TNBS), University Hospital Essen, Essen, Germany; 4 Department of Neuroradiology, Clinic Hirslanden, Zurich, Switzerland

**Keywords:** Radiation exposure, Intracranial aneurysm, Coiling, Interventional neuroradiology

## Abstract

**Purpose:**

Endovascular treatment of unruptured intracranial aneurysms (UIAs) requires a risk–benefit analysis and adherence to diagnostic reference levels (DRLs). The national DRL (250 Gy·cm^2^) is only determined for intracranial aneurysm coiling in general, including ruptured intracranial aneurysms (RIAs). This study aims to investigate the dose in the treatment of UIAs and RIAs separately.

**Methods:**

In a retrospective study design, dose area product (DAP) and fluoroscopy time (FT) were assessed for all patients undergoing intracranial aneurysm coiling between 2010 and 2021. DRL was set as the 75th percentile of the dose distribution. A multivariable linear regression analysis was performed to investigate DAP and FT for the two groups, UIA and RIA adjusted for patient age, aneurysm size, and location.

**Results:**

583 (414 females, mean age 56.5 years, 311 UIAs) are included. In the overall population, DAP (median (IQR)) is 157 Gy·cm^2^ (108–217) with a median FT of 32.7 min (IQR 24.0–47.0). Local DRL is 183 Gy·cm^2^ for UIAs and 246 Gy·cm^2^ for RIAs. After adjustment for the other variables, the UIA and RIA groups have a significant effect on both DAP (*p* < 0.001; 95% CI − 68.432 – − 38.040) and FT (*p* < 0.001; 95% CI − 628.279 – − 291.254). In general, both DAP and FT increase significantly with patient age and aneurysm size, whereas the location of the aneurysm did not significantly change neither DAP (*p* = 0.171; 95% CI − 5.537–31.065) nor FT (*p* = 0.136; 95% CI − 357.391–48.508).

**Conclusion:**

Both aneurysm size and patient age were associated with increased DAP, whereas aneurysm location did not significantly change DAP or FT. The increased dose in patients with RIAs is likely equivalent to additional diagnostic cerebral four-vessel angiography performed in this group.

## Introduction

Endovascular treatment of unruptured (UIAs) and ruptured intracranial aneurysms (RIAs) has become a standard procedure and increased significantly over the past decades [1–3]. With the recent update of the radiation protection guidelines, compliance with diagnostic reference levels (DRLs) has become more important. Therefore, a risk–benefit analysis regarding endovascular treatment is especially crucial in patients with an incidental, UIA, and demands for observance of DRLs.

Published data on radiation dose are mostly based on unselected patient cohorts, i.e., patients with both emergency and elective aneurysm treatment [4–6]. Consequently, the national DRL, in terms of dose area product (DAP), published by the Federal Office of Radiation Protection only refers to intracranial aneurysm coiling (250 Gy·cm^2^) in general and does not distinguish between UIAs and RIAs [7].

The aim of this study is to investigate the radiation dose and fluoroscopy time (FT) in patients with UIAs and RIAs with reference to aneurysm size and location, and patient age. The reported data may serve as a benchmark for the next update of DRLs in endovascular coiling of intracranial aneurysms.

## Methods

### Patient cohort

**Fig. 1:**
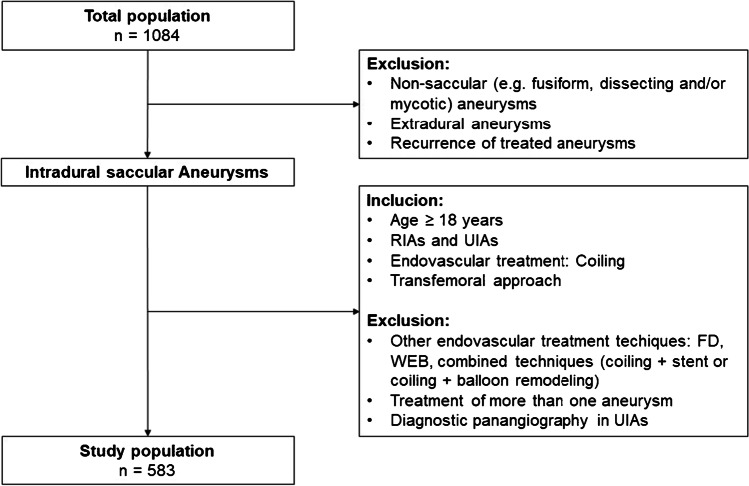
Flow chart of analyzed patient cohort with inclusion and exclusion criteria. FD, flow diverter; *n*, number; RIA, ruptured intracranial aneurysm; UIA, unruptured intracranial aneurysm; WEB, Woven EndoBridge device

This retrospective single-center study was approved by the local institutional review board (Ethics Commission of the Medical Faculty of the University of blinded, 21–10,485-BO). The internal angiographic database of the radiology department was searched for all endovascular treatments of intracranial aneurysms between January 2010 and December 2021. All inclusion and exclusion criteria were defined in the flowchart (see Fig. 1) to increase the dosimetric homogeneity of the data.

### Procedure

A transfemoral approach was used for all included procedures. To capture the dosimetric data of a standardized aneurysm coil embolization at our department, we need to distinguish between patients with an UIA and a RIA. Typically, patients with an UIA underwent diagnostic angiography extern or in-house prior to coil embolization, whereas in the endovascular treatment of patients with a RIA typically an additional diagnostic cerebral angiography of at least four-vessel before intervention was performed to rule out further aneurysms and to image patients’ vasculature anatomy.

In both patient groups, the standard angiographic workflow routinely comprises the following steps: (a) placement of the guiding catheter into the target vessel, (b) initial digital subtraction angiography (DSA) run on standard lateral and posterior/anterior projections, (c) 3D rotational angiography, (d) coil embolization of the aneurysm, and (e) final biplane DSA run.

### Angiography system

All embolizations were performed on the Allura Xper FD20/10 system (Philips Healthcare, Eindhoven, The Netherlands). The angiography system is equipped with an automatic exposure control system, one detector 20 in. with a maximum field of view (FOV) of 48 cm and one 10-in. detector with a max. FOV of 25 cm. All procedures were performed by an experienced team of neuroradiologists using a pulsed fluoroscopy mode with a frame rate of 1 and 3 pulse/s and a focus-to-skin distance from 60 to 70 cm. The Philipps system used in our study remained constant throughout the period and was always provided with the latest version of the Philipps program updates. Over time, regular quality control checks were carried out during maintenance visits to ensure a continuous, high-level system performance.

### Data and dose assessment

For each patient, the dosimetry report and imaging data were extracted from the picture archiving and communication system (PACS) and all clinical information was retrieved from the radiology information system (RIS). In detail, the following information was documented: age, sex, aneurysm size, and location, indication for the intervention (e.g., subarachnoid hemorrhage due to ruptured aneurysm), endovascular technique, fluoroscopy time (FT), and DAP. DRLs were set as the 75th percentile of the dose distribution [8]. Depending on the localization of the aneurysm, coil embolizations were assigned to the anterior and posterior circulation (see Table 1).

**Table 1:** Characteristics of 583 patients with a saccular intracranial aneurysma undergoing coil embolization

		UIA	RIA	Total
Patient characteristics	Patient number	311	272	583
	Sex	229 females (73.6%), 82 males (26.4%)	185 females (68.0%), 87 males (32.0%)	414 females (71.0%), 169 males (29.0%)
	Age, mean (range)	57.1 years (23–89)	55.8 years (22–93)	56.5 years (22–93)
	Aneurysm size, mean (range)	7.1 mm (2.0–35 mm)	6.8 mm (1.4–21 mm)	6.9 mm (1.4–35 mm)
Aneurysm localization	Anterior circulation	244 patients (78.5%)	211 patients (77.6%)	455 patients (78.0%)
	ICA	117 (37.6%)	55 (20.2%)	172 (29.5%)
	ACOM	78 (25.1%)	130 (47.8%)	208 (35.7%)
	ACA	7 (2.3%)	10 (3.7%)	17 (2.9%)
	MCA	19 (6.1%)	5 (1.8%)	24 (4.1%)
	PerA	23 (7.4%)	11 (4.0%)	34 (5.8%)
	Posterior circulation	67 patients (21.5%)	61 patients (22.4%)	128 patients (22.0%)
	BA	50 (16.1%)	32 (11.8%)	82 (14.1%)
	VA	1 (0.3%)	5 (1.8%)	6 (1.0%)
	PCA	6 (1.9%)	6 (2.2%)	12 (2.1%)
	SCA	6 (1.9%)	6 (2.2%)	12 (2.1%)
	PICA	4 (1.3%)	12 (4.4%)	16 (2.7%)
Aneurysm size	** < **5 mm 5–9 mm 10–14 mm ** ≥ **15 mm	96 (30.9%) 164 (52.7%) 36 (11.6%) 15 (4.8%)	87 (32.0%) 142 (52.2%) 29 (10.7%) 14 (5.1%)	183 (31.4%) 306 (52.5%) 65 (11.1%) 29 (5.0%)

*ACOM* anterior communicating artery; *BA* basilar artery; *ICA* intradural segments of the internal carotid artery, including posterior communicating artery and carotid T; *MCA* middle cerebral artery; *PCA* posterior cerebral artery; *PerA* pericallosal artery; *PICA* posterior inferior cerebellar artery; *RIA* ruptured intracranial aneurysm; *SCA* superior cerebellar artery; *UIA* unruptured intracranial aneurysm; *VA* vertebral artery

### Statistical analysis

Statistical analysis was performed using Statistical Package for Social Sciences v. 28.0. (SPSS Inc., New York, USA). Data were initially assessed for normality by applying the Shapiro–Wilk test and Kolmogorov–Smirnov. We performed a multivariable linear regression analysis for the dependent variables DAP and FT as a function of patient age, aneurysm size, location, and a binary variable for the type of aneurysm, ruptured or unruptured. The size of the aneurysm was entered as a continuous variable. A *p* value of < 0.05 was considered statistically significant.

## Results

### Patient cohort

In our retrospective study, 1084 endovascular treatments of intracranial aneurysms were performed between January 2010 and December 2021, of which 583 coil embolizations of intracranial aneurysms could be included for evaluation (see Fig. 1). The mean age was 56.5 years (range 22–93 years), and 71.0% (414/583) of patients were female. Slightly more than half of the patients were treated with an UIA (53.3%; 311/583). On average, the aneurysms were 6.9 mm in size (median 6 mm), and the maximum diameter varied between 1.4 and 35 mm. In about half of the patients (52.5%), the aneurysm was between 5 and 9 mm in size. Four hundred fifty-five (78.0%) of the aneurysms were located in the anterior circulation and 128 (22.0%) in the posterior circulation. Patient characteristics are described in Table 1.

### Radiation dose and fluoroscopy time

**Fig. 2:**
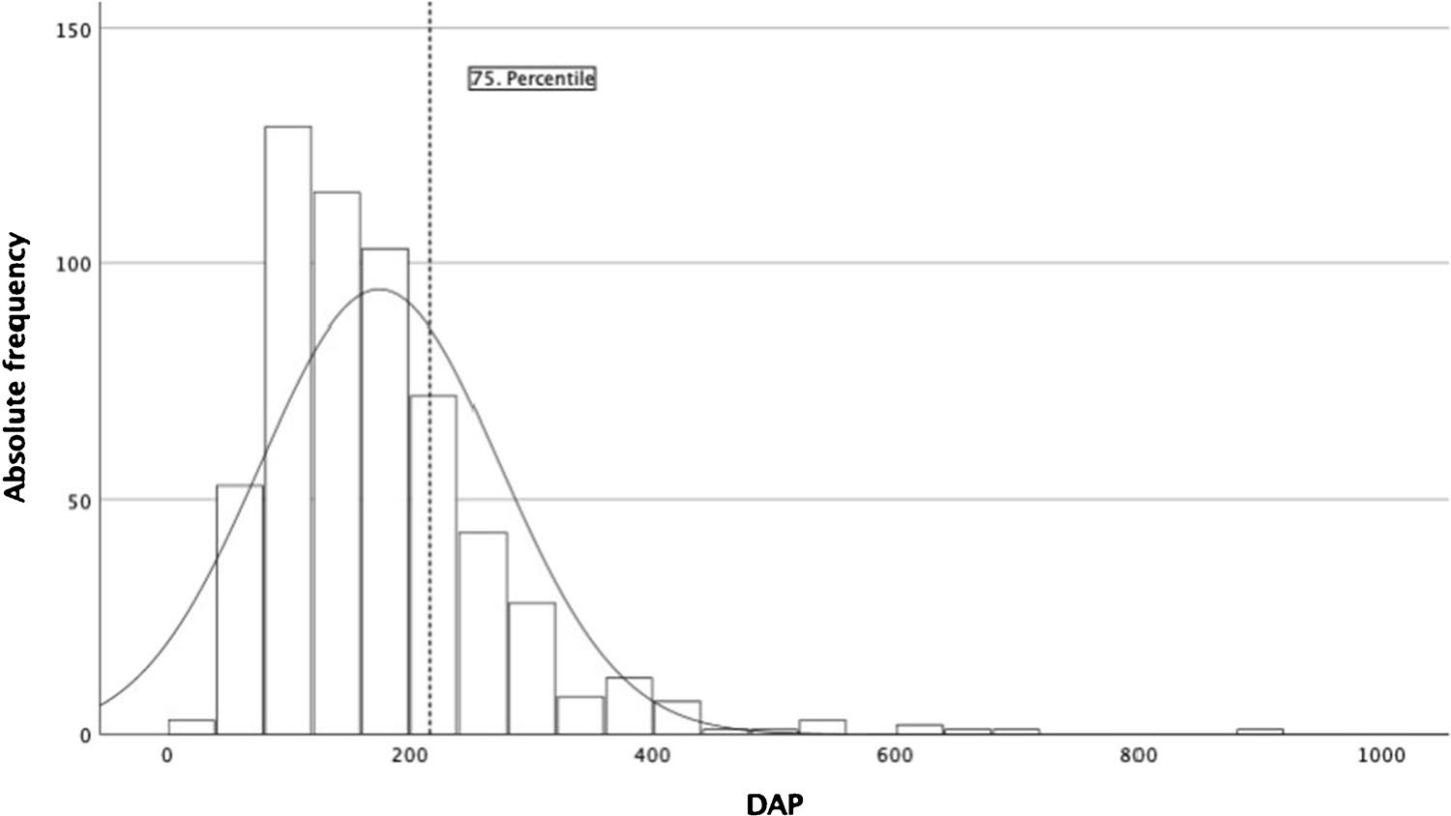
Histogram distribution of dose area product (DAP in Gy∙cm^2^) for coil embolization of intracranial aneurysms in the overall population; curve highlighting distribution graph

**Fig. 3:**
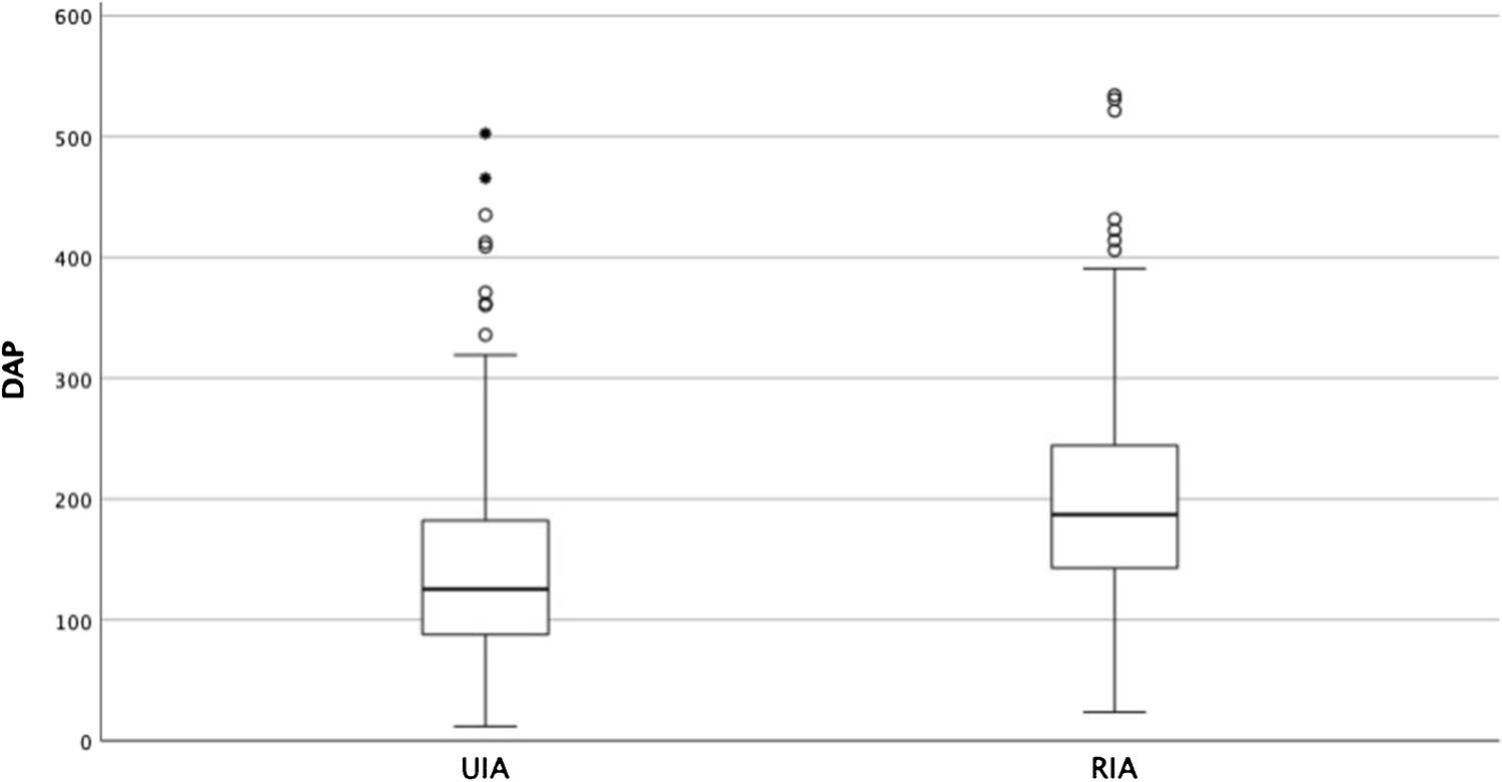
Dose area product (DAP) during coil embolization of unruptured (UIA) and ruptured intracranial aneurysms (RIA). Points show outliers outside the Tukey whiskers. Four outliers for the group UIAs and one outlier for the group RIAs, each above 600 Gy·cm^2^ are not depicted in the graph

**Fig. 4:**
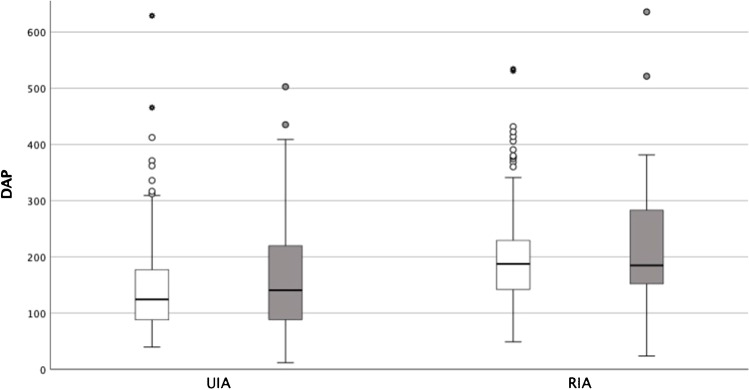
Dose area product (DAP) during coil embolization of unruptured (UIA) and ruptured intracranial aneurysms (RIA) subdivided by anterior (white) and posterior circulation (gray). Points show outliers outside the Tukey whiskers. Three outliers for the group UIAs anterior circulation above 650 Gy·cm^2^ are not depicted in the graph

The dosimetric data is summarized in Tables 2 and 3 and illustrated in Figs. 2, 3, and 4. In the overall population, DAP (median (IQR)) is 157 Gy·cm^2^ (108–217) with a median (IQR) FT of 32.7 min (24.0–47.0) (Fig. 2). Local DRL is 183 Gy·cm^2^ for UIAs and 246 Gy·cm^2^ for RIAs. The multivariable linear regression analysis for DAP and FT includes the terms age of the patient, location of aneurysm, size, and a binary variable for the type of aneurysm, ruptured or unruptured. After adjustment for the other variables, the UIA and RIA groups have a significant effect on both DAP (*p* < 0.001; 95% CI − 68.432 – − 38.040) and FT (*p* < 0.001; 95% CI − 628.279 – − 291.254) (Fig. 3). Both DAP and FT increase significantly with patient age and aneurysm size (see Table 2), whereas the location of the aneurysm did not significantly change neither DAP (*p* = 0.171; 95% CI − 5.537–31.065) nor FT (*p* = 0.136; 95% CI − 357.391–48.508) (Fig. 4).

**Table 2:** Multivariable linear regression analysis for dose area product and fluoroscopy time as a function of patient age, aneurysm size, location, and type of aneurysm

	Predictors	Std. error	*p* value	95% CI
DAP	Age (in years)	0.316	0.025	0.089–1.330
	Size (in mm)	1.003	< 0.001	2.818–6.757
	Location (anterior/posterior circulation)	9.318	0.171	− 5.537–31.065
	Type of aneurysm (ruptured /unruptured)	7.737	< 0.001	− 68.432– − 38.040
FT	Age (in years)	3.504	< 0.001	16.143–29.908
	Size (in mm)	11.121	< 0.001	28.706–72.391
	Location (anterior/posterior circulation)	103.331	0.136	− 357.391–48.508
	Type of aneurysm (ruptured /unruptured)	85.797	< 0.001	− 628.279– − 291.254

*DAP* dose area product [Gy·cm2]; *FT* fluoroscopy time [sec]. DAP: *R*^2^ = 0.116, corr. *R*^2^ = 0.109; *F*(4578) = 18.875, *p* < 0.001

FT: *R*^2^ = 0.136, corr. *R*^2^ = 0.131; *F*(4578) = 22.839, *p* < 0.001

**Table 3:** Radiation dose and fluoroscopy time of aneurysm coiling in patients with unruptured and ruptured intracranial aneurysms

	DAP [Gy·cm^2^]		FT [min]
	Mean	Median (IQR)	Median (IQR)
Total	175.2	157.0 (108.3–216.9)	32.7 (24.0–47.0)
UIA	151.2	125.5 (87.9–182.6)	29.2 (22.2–40.9)
RIA	202.5	187.2 (142.7–245.6)	36.3 (26.7–52.3)

*DAP* dose area product, *FT* fluoroscopy time, *RIA* ruptured intracranial aneurysm, *UIA* unruptured intracranial aneurysm

## Discussion

The present study provides detailed dosimetric data of aneurysm coiling in patients with UIAs and RIAs. To our best knowledge, this is the first study comparing DRL from patients with RIA and UIA. The national radiation protection guidelines only provide DRLs for cerebral aneurysm coiling (DAP 250 Gy·cm^2^) in general and do not distinguish between the indication for aneurysm treatment (elective or emergency) [7]. Therefore, the reported data may be valuable in the next update of DRLs for intracranial aneurysm coiling.

Based on the Euratom Basic Safety Standards directive and the ICRP 135 publication, the DRL concept is well established and the DRL catalog was recently expanded, especially with regard to regularly used interventional X-ray procedures [9, 10]. In general, it is recommended to collect several dose-related parameters (e.g., DAP and fluoroscopy time) and to define national DRL based on the 75th percentile of the distribution of the DAP of a specific radiological procedure [8, 9, 11, 12]. Because of the high individual variability within an interventional X-ray procedure, radiation dose metrics of more than 50 specific procedures should be collected to determine DRLs for a single-center [13]. This allows equipment operators to determine if the radiation dose is within the normal range of a dose distribution or if dose optimization may be required. According to the linear no-threshold model, any exposure to ionizing radiation is potentially harmful and bears the risk of developing radiation-induced cancer, regardless of the amount of dose [14, 15]. Therefore, the indication for each patient must be carefully determined and every radiation application optimized according to the ALARA (as low as reasonably achievable) principle [16].

In the present study, we included 583 procedures and calculated a total mean and median (IQR) DAP of 175 Gy·cm^2^ and 157 (108–217) Gy·cm^2^. In relation to the literature, our local DRL for aneurysm coiling (217 Gy·cm^2^) was slightly below the previously updated national DRL for coiling (250 Gy·cm^2^) in 2018 [8]. In detail, the 75th percentile was 183 Gy·cm^2^ for the coiling of UIAs and 246 Gy·cm^2^ for the coiling of RIAs. The reported significant difference in radiation exposure between the treatment groups is probably due to the additional diagnostic cerebral four-vessel angiographies performed during the same procedure in patients with RIA to exclude further aneurysms. In this context, Acton et al. published a median DAP of 74 Gy·cm^2^ for a cerebral four-vessel angiography [17]. However, patients with an UIA already underwent diagnostic angiography extern or in-house prior to coil embolization, resulting in an appropriate amount of DAP.

Forbrig et al. [18] provided dosimetric data in patients with a saccular UIA according to different endovascular treatment techniques. In the small subgroup for coiling (*n* = 23), the published DRL (130 Gy∙cm^2^) was slightly lower compared to our study (183 Gy·cm^2^). However, due to a different procedural in-house management, several aneurysm locations were excluded in this study and thus dosimetric data cannot be fully compared.

In a multicenter study, Ihn et al. reported on the radiation exposure during diagnostic and therapeutic procedures for intracranial aneurysms using a modern biplane angiography system [19]. The published DRL of 271.0 Gy·cm^2^ for endovascular treatment of intracranial aneurysms was higher compared to our study. However, the authors did not distinguish between the embolization technique and the UIA and RIA subgroups.

Several studies have reported on radiation exposure and FT during neurointerventional procedures with modern biplane angiography systems, e.g., mechanical thrombectomy [20, 21] and endovascular treatment of intracranial dural arteriovenous fistulae [22, 23] or carotid-cavernous fistula [24]. The aim is to raise awareness of the radiation dose and, in the long term, to optimize the modification of angiography systems and therapeutic techniques. In our study, both DAP and FT increase significantly with patient age and aneurysm size. The positive correlation of radiation dose and both aneurysm sizes as well as patient age was also reported by other authors [22, 25]. Depending on the size of the aneurysm, the endovascular treatment approach often becomes more complex, and the anatomic approach in elderly patients is also often more challenging, as tortuosity of the vessels is often associated with the increasing complexity of treatment, which affects radiation exposure. However, additional parameters such as the type of aortic arch or carotid stenosis were not taken into account.

Considering the aneurysm location, our study is consistent with previous studies that have shown that DAP and FT are not significantly different when comparing the aneurysm locations [17, 22]. However, in our study, several rare aneurysm locations (e.g., superior cerebellar artery or posterior cerebral artery, see Table 1) were included and assigned to the anterior and posterior circulation. Therefore, the dosimetric data reported in the present study can only serve as an approximation with regard to aneurysm location and further multicenter studies are the next necessary step to obtain definitive results.

As shown by other authors [19, 25, 26], radiation dose metrics of interventional X-ray procedures are influenced by multiple factors (e.g., the complexity of procedures, endovascular treatment technique, experience of the medical staff, and angiographic system settings). For this reason, we established some inclusion and exclusion criteria to increase the dosimetric homogeneity of data collection in our study (Fig. 1).

In addition to the retrospective and single-center study design, the following limitations should be noted. First, all intracranial aneurysm embolizations were performed with only one angiographic system from a single manufacturer (Philips Healthcare). Therefore, the dose values obtained may differ from those of other angiography devices and sites. Second, all procedures were performed by an experienced team of neuroradiologists, but at our university hospital, young physicians are also trained. Thus, in terms of dosimetric data, our findings may suggest higher doses than can be achieved. Third, as shown in the flow chart (Fig. 1), we excluded some procedures (e.g., using other endovascular treatment techniques); consequently, our study does not represent the entire spectrum of endovascular treatment approaches. However, in our opinion, the selected study population from the last decade can serve as a representative cohort of patients with saccular intracranial aneurysms and comparative studies, especially with regard to the comparison of UIA and RIA, are lacking. Further studies on this topic will be needed in the future, as the impact of evolving technologies (e.g., better guiding catheters, better proximal support) and increasing experience in using these technologies may have an impact on radiation dose and FT.

## Conclusion

In conclusion, this study provides dosimetric data in the field of intracranial aneurysm coiling of patients with UIAs and RIAs with reference to aneurysm size and location, and patient age. Radiation dose, in terms of DAP, and FT for UIAs are significantly lower compared to RIAs. However, the increased dose in patients with RIAs is likely equivalent to additional diagnostic cerebral four-vessel angiography. For this reason, we do not believe it is necessary to establish separate DRLs for intracranial aneurysm coiling in patients with UIA and RIA. In general, both patient age and aneurysm size were associated with increased DAP and FT, whereas aneurysm location did not significantly change neither radiation dose nor FT.

## Data Availability

The authors declare data availability upon explicit request.
